# Regulation of chronic neuroinflammation through dietary herbal products

**DOI:** 10.3389/fnut.2025.1487786

**Published:** 2025-04-09

**Authors:** Kumar M. R. Bhat, Raghavendra Upadhya, Shripathi Adiga, S. E. Praveen Kumar, S. D. Manjula, Nanda Acharya, Hari H. Subramanian, Dinesh Upadhya

**Affiliations:** ^1^Department of Anatomy, Kasturba Medical College, Manipal, Manipal Academy of Higher Education, Manipal, Karnataka, India; ^2^Manipal Centre for Biotherapeutics Research, Manipal, Manipal Academy of Higher Education, Manipal, Karnataka, India; ^3^Department of Ayurveda, Centre for Integrative Medicine and Research, Manipal, Manipal Academy of Higher Education, Manipal, India; ^4^Department of Pharmacology, Manipal Tata Medical College, Jamshedpur, Manipal Academy of Higher Education, Manipal, India; ^5^Department of Physiology, Kasturba Medical College, Manipal, Manipal Academy of Higher Education, Manipal, Karnataka, India; ^6^Department of Neurosurgery, Mayo Clinic, Jacksonville, FL, United States; ^7^Neuronano AB, Valencia, CA, United States; ^8^Centre for Molecular Neurosciences, Kasturba Medical College, Manipal, Manipal Academy of Higher Education, Manipal, Karnataka, India

**Keywords:** neurological disorders, neuroinflammation, dietary herbs, cytokines, animal models

## Abstract

Chronic neuroinflammation is a consequence of disease pathogenesis underlying neurological disorders at large. While the immune response that triggers inflammatory signaling cascades is unresolved, its progression could cause functional damage to neurons and glial cells, including astrocytes, microglia, and oligodendrocytes. Controlling neuroinflammatory signaling at the early stage of disease pathogenesis is critical to prevent irreversible tissue necrosis. While the application of anti-inflammatory drugs is standard practice, their protracted use is known to cause gastrointestinal injuries, further enhancing the risk of cardiovascular, renal, liver, and lung diseases. Several medicinal herbs and herbal products with anti-inflammatory potential could be effective substitutes. This review aims to identify the preclinical data from important dietary herbal products that have demonstrated anti-neuroinflammatory efficacy in several animal models. The reviewed dietary herbal products are sourced from *Bacopa monnieri, Centella asiatica, Emblica officinalis, Piper nigrum, Zingiber officinale, Punica granatum, Mucuna pruriens, Clitoria ternatea, Moringa oleifera, Phoenix dactylifera* and *Curcuma longa*. This review is based on emphatic data from these products demonstrating the significant anti-neuro-inflammatory potential that could probably reduce neuroinflammatory signaling in a neurological disorder and promote brain health and well-being. Abundant scientific evidence shows that critical proinflammatory cytokines in the brain, such as tumor necrosis factor-alpha (TNF-*α*), interleukin-1 beta (IL-1β), interleukin-six (IL-6), could be controlled through regular consumption of such dietary herbal products without debilitating side effects for their disease-modifying impacts.

## Introduction

Chronic neuroinflammation is associated with almost every neurological, cerebrovascular, autoimmune, metabolic disorder, and brain tumor. Although the etiology may be intrinsic or extrinsic, the site of origin is unknown, and the role played by neuroinflammation in their pathogenesis is enormous. An unresolved immune response triggered by different stimuli is believed to be the earliest cause of initiation of the inflammatory cascade. This could modify into a chronic neuroinflammatory stage, probably leading to tissue damage through various pathways (1). Progression of neuroinflammation could cause functional damage to neurons, astrocytes, microglia, oligodendrocytes, etc. These changes interfere with cellular mechanisms such as cell survival, proliferation, dendritic arborization, blood–brain barrier integrity, synaptic plasticity, and neurotropic signaling. Additionally, prolonged neuroinflammation may also induce functional alteration including sensory, motor and cognitive functions, etc. (2, 3).

Controlling the progress of the neuroinflammatory cascade at the earliest stage of the disease pathogenesis through medical interventions is important to prevent the progression into a stage of irreversible tissue damage. Also, chronic brain inflammation could elevate systemic inflammation levels, leading to further organ damage. Therefore, controlling the chronic neuroinflammation at the earliest could prevent their structural and functional damage (4–6).

Preclinical studies demonstrated significant beneficial effects of various anti-inflammatory drugs in animal models as a primary treatment or adjuvant therapy for disease conditions (3). Consideration for adding an anti-inflammatory medication along with the primary treatment could decrease disease progression in many organ systems, even in human studies (7). Nonsteroidal anti-inflammatory drugs have been used clinically over the years to control inflammation. Major classes of anti-inflammatory drugs used include nonsteroidal anti-inflammatory drugs (NSAIDs) and steroids, primarily corticosteroids. Commonly used NSAIDs include acetylated and nonacetylated salicylates, propionic acids, enolic acids, anthranilic acids, and selective cyclooxygenase-2 (COX-2) inhibitors. However, several of these NSAIDs cause gastric mucosal and small bowel injuries, increasing the risk of cardiovascular diseases, renal injury, liver damage, intracerebral hemorrhage, respiratory tract inflammation, infection, etc. (8). Apart from such adverse effects, preventable adverse NSAID reactions also result in 30% of hospital admissions (9).

While the serious side effects of the long-term use of several classic antiinflammatory drugs are established, advanced biological agents are unaffordable for their continuous use. Thus, several patients pursue lifestyle and naturopathic remedies (7). Several plants, including herbs, shrubs, trees, and their products, are well-established for their nutritional value. Including such sources in the diet could reduce the burden of neuroinflammation without significant side effects and affordability issues for long-term use as they are natural sources.

In the literature, dispersed information is available from herbal products and bioactive components demonstrating anti-inflammatory and disease-modifying activity for various disorders. Earlier reviews focused on the anti-neuroinflammatory activity of specific bioactive compounds rather than whole herbal products. This may be because the anti-neuroinflammatory activity could be attributed to those specific compounds (10–15). Most herbal products are not used in regular diets, and individual bioactive components are not dietary. Comprehensive reviews on regulating chronic neuroinflammation through dietary herbal products are unavailable in the literature. Also, there is a lack of comprehensive reviews on using common dietary herbs in crude forms that are easy to use as a dietary source for preventing neuroinflammation. We believe such reviews are unavailable due to the lack of a large pool of studies for any specific dietary herbal product. In contrast, such reviews are available for individual bioactive components from dietary herbal products, such as curcumin (16).

The present work aims to review common dietary herbs, mainly in extracted forms. This review discussed only the use of whole plant/plant parts extracts as they are usually used in diet or traditional medicine. Also, the present review focused only on using reliable data from *in vivo* studies, while cell culture data on the effects of these products were not included. While the combination of herbal products may have added benefits, such studies are not included in this review as they further complicate the identification of the action source. For the literature search, we used the terms “dietary herbal products AND anti-inflammatory,” dietary herbal products AND neuroprotection,” “dietary herbal products AND brain,” “dietary herbs and AND brain,” “dietary herbs AND anti-inflammatory,” dietary herbal extracts AND anti-inflammatory “and several other matching terms. Our search included articles from PubMed, Scopus, Web of Science, and some articles from Google Scholar that demonstrated detailed methodology.

The selection of dietary herbal products was based on their demonstration of various health benefits via regulating neuroinflammation as one of the significant mechanisms of action. We selected studies that demonstrated benefits in clinical studies and animal models of neurological disorders. Also, the study was restricted to the herbal dietary products demonstrating their neuroinflammation regulatory role in at least two independent studies. This helped us to narrow down the number of dietary herbal products from the large pool as for several products, a single article report is available. Based on the abundance of work done and data available, we have shortlisted the following dietary herbal sources commonly used in India as we know their authenticity as a dietary product: *Bacopa monnieri, Centella asiatica, Emblica officinalis, Piper nigrum, Zingiber officinale, Punica granatum, Mucuna pruriens, Clitoria ternatea, Moringa oleifera, Phoenix dactylifera and Curcuma longa* for the analysis.

Earlier reviews focused on the anti-neuroinflammatory activity of specific bioactive compounds rather than whole herbal products. This review is unique, as we have comprehensively reviewed common dietary herbs in crude forms that are easy to use as a dietary source for preventing neuroinflammation. Our review does not cover the anti-inflammatory effects of specific isolated bioactive compounds from these plants. In contrast, the impact of our selected herbal dietary product may be attributed to these compounds. As commonly used in the diet, such herbs are proven nondetrimental to health while providing exceptional benefits for controlling neuroinflammation. Based on this review, we believe further research will be undertaken worldwide to establish their benefits through well-planned molecular studies. This could promote herbal dietary products for controlling chronic neuroinflammation, commonly associated with neurological disorders.

### Bacopa monnieri

**Figure 1:**
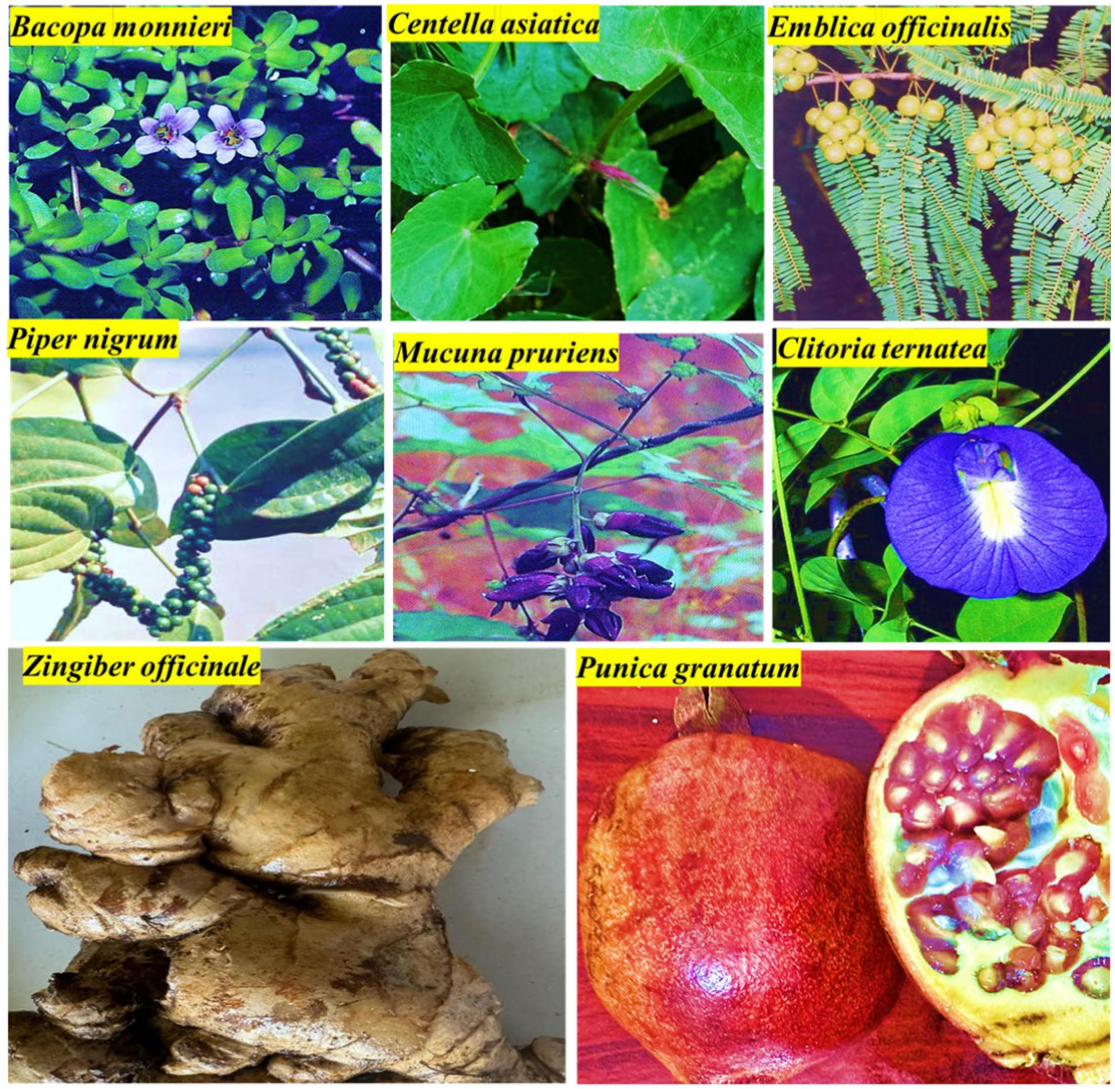
Representative images of *Bacopa monnieri*, *Centella asiatica*, Emblica officinalis, *Piper nigrum*, *Mucuna pruriens* and *Clitoria ternatea*, *Zingiber officinale*, and *Punica granatum*.

**Figure 2:**
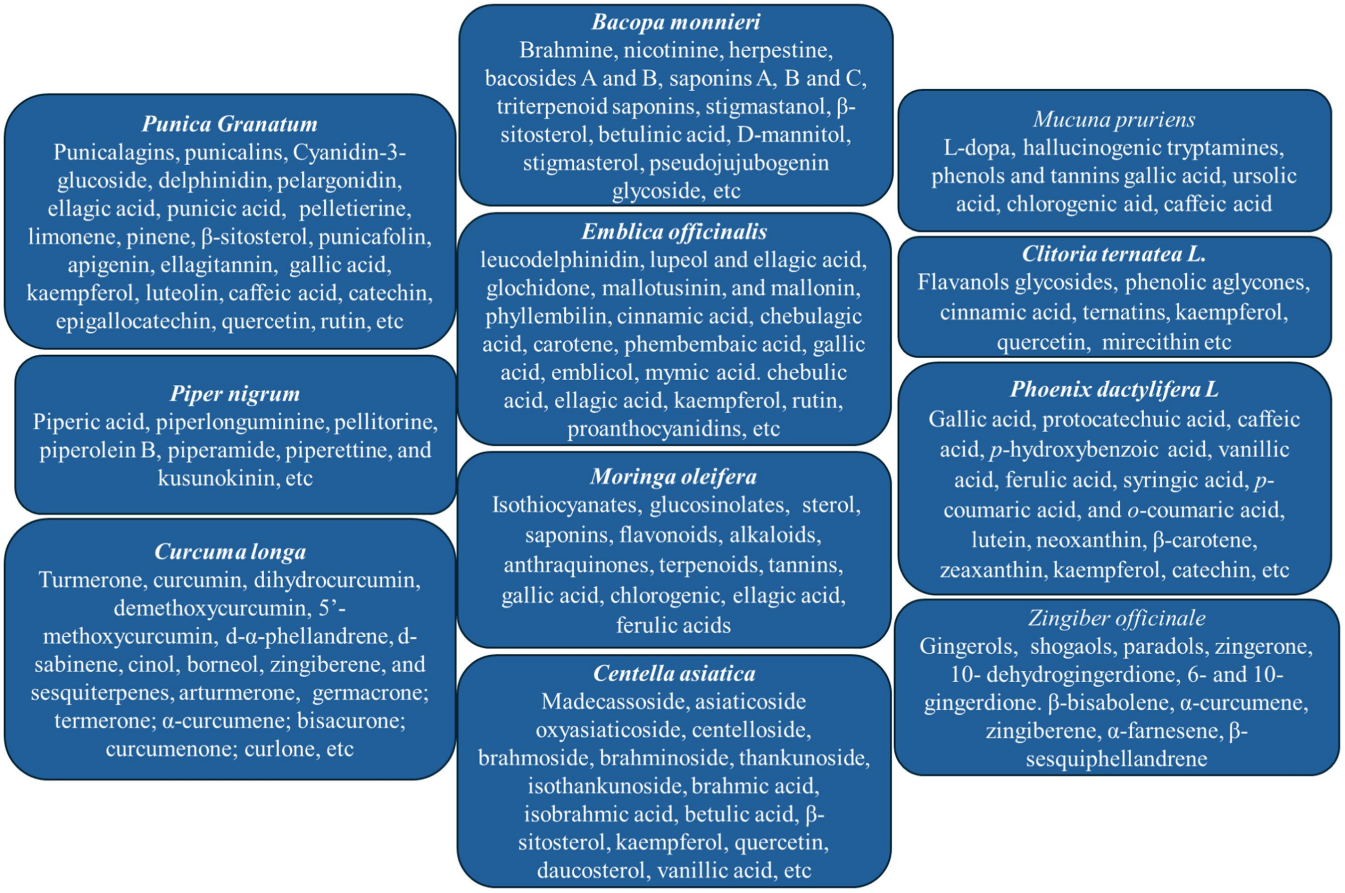
Major bioactive components of *Bacopa monnieri* (17, 18), *Centella asiatica* (26), *Zingiber officinale* (33–35), *Punica granatum* (43, 44), Emblica officinalis (50, 51), *Piper nigrum* (54, 55), *Mucuna pruriens* (58), *Clitoria ternatea* (65, 66), *Moringa oleifera* (70), *Phoenix dactylifera* (82–84) and *curcuma longan* (90).

*Bacopa monnieri* (BM, Figure 1), commonly known as Brahmi, is a perennial, creeping herb found mainly in southern and Eastern India. BM is also found in other parts of Asia, Australia, Africa, Europe, and North and South America. Bioactive constituents of BM include bacosides A and B, brahmine, polyphenols, herpestine, flavonoids, terpenoids, and proteins (17, 18) (Figure 2). Apart from its dietary use, it has been extensively used to treat neurological disorders in traditional medicine and has been shown to have anti-diabetic, hepatoprotective, anti-cancer, anti-metastatic, anti-fatigue and vasodilator effects in several experimental studies (19, 20).

In an open-labeled human study, BM (two capsules/day containing 320 mg of dried extract of BM whole plant) was supplemented to 35 cognitively healthy, aging (60-78-year-old) control subjects over 3 months to identify its impact on safety and cognitive aging and molecular markers. Supplementation of BM demonstrated a good safety profile. Evaluation of subjects with Montreal Cognitive Assessment showed significant improvement in the delayed-recall subscale. BM supplementation demonstrated reduced levels of phosphorylated p65 nuclear factor kappa-B (NF-κB) in serum, indicating suppression of aging-related inflammation. This supplementation increased the cAMP response element binding protein (CREB) phosphorylation with improved synaptogenesis, leading to improved cognitive ability (21).

In a rotenone-induced model of Parkinson’s disease (PD), TNF-*α*, IL-1β, IL-6 and macrophage inflammatory protein-1 beta (MIP-1b) along with α-synuclein were shown to be significantly reduced in hippocampus, substantia nigra, and striatum region and slight changes in cortex and brain stem region of animals with oral supplementation of (40 mg/kg body weight/for 4 weeks) BM. Interestingly, pre-treatment of BM showed better results than co-or post-treatment (22). In the propionic acid-induced rat model of autism spectrum disorder (ASD), oral administration of 250 mg/kg and 500 mg/kg hydroalcoholic extract of BM for 28 days is found to dose-dependently reduce the TNF-𝛼 in brain tissues along with cognitive improvements (23). In the Valproic acid-induced autism spectrum disorder model of rats, oral administration of 20, 40, and 80 mg/kg of BM methanolic extract for 20 days decreased the inflammation in the hippocampus and prefrontal cortex by decreasing the IL-1β, IL-6, and TNF-*α* levels dose-dependently. BM also attenuated the oxidative stress in these regions and improved the developmental parameters, social interactions, self-grooming, memory and learning, anxiety-like behavior, and motor coordination in valproic acid-induced rats, along with a reduction in the neuronal injuries in the hippocampus and prefrontal cortex (24).

From the available studies, it is evident that BM increases CREB phosphorylation and reduces TNF-*α*, IL-1β, IL-6, NF-κB and MIP-1b, in different brain regions, which results in reduced neuroinflammation that also controls disease progression.

### Centella asiatica

*Centella asiatica* (CA, Figure 1) is a clonal, perennial herbaceous creeper, a tropical, medicinal plant from the Apiaceae family from Southeast Asian countries such as India, Sri Lanka, China, Indonesia, and Malaysia, as well as South Africa and Madagascar [reviewed in Orhan (25)]. It is commonly known as *mandukparni*, Indian pennywort, or *jalbrahmi* and is used for multiple daily food preparations.

The most bioactive constituents of CA are triterpenoids, amino acids, and essential oils. Among them, the most important compounds are triterpene glycosides (saponins), which include asiaticoside and madecassoside with their respective aglycones (sapogenins), asiatic acid and madecassic acids. These constituents are shown to be responsible for CA’s biological activities/medicinal properties. In addition, there are several other constituents which include oxyasiaticoside, centelloside, brahmoside, brahminoside, thankunoside, isothankunoside, brahmic acid, isobrahmic acid, betulic acid, *β*-sitosterol, hexacosanol octanoate, kaempferol, quercetin, daucosterol, vanillic acid, and succinic acid. However, madecassoside and asiaticoside are present in higher concentrations and are considered the most important biomarker of CA [reviewed in Tan et al. (26), Figure 2].

CA is beneficial in treating leprosy, lupus, varicose ulcers, eczema, psoriasis, diarrhea, fever, amenorrhea, and diseases of the female genitourinary tract, in addition to its effect on relieving anxiety and improving cognition (27). Several studies show its neuroprotective effects, mainly mediated through its anti-neuroinflammatory activities. Table 1 explains the various studies that show the anti-neuroinflammatory properties of CA. It is evident from these studies that CA demonstrates its anti-neuroinflammatory activities in the brain through modulating TNF-*α*, IL-1β, suppressing the translocation of NF-kB p65, and activation of protein kinase B (Akt) and extracellular signal-regulated kinase 1/2 (ERK1/2) pathways in several disease models such as sleep-deprived induced anxiety, Huntington’s disease and hypoxia-induced ischemic stroke (28–30). Oral administration of 200 mg/kg of CA for 14 days reduces the plasma levels of IL-6, IL-1β, TNF-α, and interferon-gamma (IFN-*γ*). It increases the level of monocyte chemoattractant protein-1 (MCP-1) in the lipopolysaccharide (LPS) induced neuroinflammation model of rats (31). It also increases the anti-inflammatory cytokine interleukin-10 (IL-10), in the hypoxia-induced Zebrafish brain (30).

**Table 1:** The anti-neuroinflammatory activity of *Centella asiatica.*

Dietary source	Study model	Molecular/biochemical analysis	Functional benefits	Ref
*Centella asiatica* ethanolic *e*xtract (150 and 300 mg/kg b.w/day for 8 days)	Sleep-deprived mouse model	Reduces the TNF-α and AchE activity in brain homogenate	Reduces anxiety-like behavior Improves locomotor activity Reduces body weight	(28)
*Centella asiatica* hydroalcoholic extract (80 and 100 mg/L/ extract for 28 days)	3-Nitropropionic acid-induced Huntington’s disease model in zebrafish	Inhibits AChE activity and nitrite levels in the brain Reduces TNF-γ and IL-1β levels in the brain tissue	Inhibits progressive neuronal damage Improves cognition and locomotor activity	(29)
*Centella asiatica* hydroalcoholic extract (20 mg/L extract for 12 days) with intermittent fasting	Hypoxia-induced aging and age-related neurodegeneration zebrafish model	Reduces the TNF-α and IL-1β along with an increase in IL-10 Modulates the AMPK, MAPK, GSK-3β, Nrf2 expression	Inhibits neuroinflammation and oxidative stress Improves mitochondrial enzyme activities Improved neuronal cell survival Improves neurobehavior	(30)
*Centella asiatica* ethanolic extract (200 mg/kg extract for 14 days)	LPS-induced rat model of neuroinflammation	Reduces the plasma levels of IL-6, IL-1β, TNF-α, IFN-γ Increases the plasma level of MCP-1	It improves spatial memory and learning Decreases systemic inflammation Improves peripheral anti-inflammatory activity	(31)

In summary, CA demonstrates its anti-neuroinflammatory activities in the brain through modulating TNF-*α*, IL-1β, IL-6, and IFN-γ, suppressing the translocation of NF-kB p65 and increasing IL-10 and MCP-1 and activating Akt and ERK1/2 pathways.

### Zingiber officinale

Ginger, scientifically known as *Zingiber officinale* (Figure 1), belongs to the family Zingiberaceae and is a part of several dietary preparations. It is one of the multifunctional herbs extensively used worldwide, and it has several medicinal, nutritional, and ethnomedical values, in addition to its use as a spice and flavoring agent. It is an essential plant in many traditional medicines worldwide, such as Ayurveda, Siddha, Chinese, Arabian, and African medicines. It has generally been used to cure various diseases such as nausea, vomiting, asthma, cough, palpitations, inflammation, dyspepsia, loss of appetite, constipation, indigestion, and pain (32). Ginger is native to tropical Asia and cultivated in many parts of the world with tropical climates, such as Australia, Brazil, China, India, Jamaica, West Africa, and parts of the United States (33).

There are many constituents in ginger, and they depend on the place of origin, weather, freshness, etc. The intense nature of the ginger is mainly due to gingerol. Other gingerol analogs, such as shogaols, paradol, and zingerone, are also found in high concentrations in this rhizome. The majority of ginger pharmacological activities are due to gingerol and shogaol. In addition, there are many phenylalkylketones, including 6-gingerol, 8-gingerol, 10-gingerol, 6-shogaol, 8-shogaol, 10-shogaol and zingerone. Further, 6-paradol, 6-and 10-dehydrogingerdione and 6-and 10-gingerdione are also present in the ginger [reviewed in Ajanaku et al. (33), Mishra et al. (34), Torkzadeh-Mahani et al. (35), Figure 2].

Table 2 demonstrates the neuroprotective role of *Zingiber officinale* via its anti-neuroinflammatory properties. Oral administration of 100 mg/kg ginger ethanolic extracts for 6 days inhibits inflammation in the nucleus accumbens. It reduces oxidative stress proteins, p38 mitogen-activated protein kinase (p38 MAPK) and glial fibrillary acidic protein (GFAP) activity in the morphine-induced neuroinflammation model of rats (35). It reduces NF-κB and TNF-*α* mRNA in the amygdala of rats in the spinal nerve ligation model of neuropathic pain at 0.375% gingerol-enriched ginger in their diet for 4 weeks (36). Ginger is shown to reduce nitric oxide (NO) and TNF-α in brain tissue of monosodium glutamate-induced neurotoxic model of rat when 0.5 and 1% of ginger is added to their diet for 6 weeks (37); reduces NF-κB, TNF-α, and IL-1β in the hippocampus of ethanol-induced neurotoxicity model of rat administered with 1 g/kg dose of ginger orally for 28 days (38). This treatment also reduces interleukin-17 (IL-17) and IFN-*γ* in the spleen and decreases the NO level in the serum demonstrating reduced neuroinflammation in MOG35-55 and pertussis toxin-induced experimental autoimmune encephalomyelitis model of rats subjected to 300 mg/kg of intraperitoneal administration of ginger hydroalcoholic extract for 21 days (39); inhibits NO, acytlecholine esterase (AChE), TNF-*α*, NF-κB, IL-1β and IL-6 in brain tissues of mercury chloride-induced neurotoxicity model of rats with oral administration of 100 mg/kg of the 6-gingerol-rich fraction of *Zingiber officinale* for 14 days (40). Further, it reduces phosphorylated ERK and histone deacetylase 1 (HDAC1) protein levels, inhibits NF-κB signaling activation and reduces IL-1β, TNF-*α* and IL-6 (41); dose-responsive inhibition of expression of GFAP, a cluster of differentiation 11b (CD11b), ionized calcium-binding adapter molecule-1 (IBA-1), TNF-α, NF-κB, and IL-1β in the amygdala, frontal cortex and hippocampus in neuropathic pain models (42) in neuropathy models, demonstrating significant anti-neuroinflammatory activity.

**Table 2:** The anti-neuroinflammatory activity of *Zingiber officinale.*

Dietary source	Study model	Molecular/biochemical analysis	Functional benefits	Ref
Ginger ethanolic extract (100 mg/kg of extract for 6 days)	Chronic morphine-induced neuroinflammation in rats	Inhibits the protein expression of p38 MAPK and GFAP in the rat nucleus accumbens	Reduces the neuroinflammation	(35)
Gingerol-enriched ginger extract (0.375% extract in the diet for 4 weeks)	Spinal nerve ligated neuropathic pain model of rats.	Inhibited the overexpression of NF-κB and TNF-α mRNA in the amygdala	Alleviated pain induced greater mechanosensitivity, emotional responsiveness, and spontaneous pain	(36)
Ginger rhizome powder (0.5 and 1% of ginger powder in diet for 6 weeks)	Monosodium glutamate-induced excitotoxic neurodegenerative model of rats	Reduced the levels of NO and TNF-α in brain tissue Improves the GABA level in brain tissue	Protects the cortical neurons from neurodegenerative changes Prevents vascular congestion in the cerebral cortex	(37)
Ginger powder (1 g/kg of powder for 28 days)	Ethanol-induced neurotoxic model of rats	Reduces NF-κB, TNF-α, and IL-1β levels in hippocampal tissues Inhibits the overexpression of GABA-A and NMDA receptors in the hippocampus	Reduces the ethanol-induced cognitive deficits Prevents the degenerative changes in the Purkinje cells of the hippocampus	(38)
Ginger hydroalcoholic extract (Intraperitoneal injection of 300 mg/kg of extract for 21 days)	MOG35-55 and pertussis toxin-induced experimental autoimmune encephalomyelitis (multiple sclerosis) model of mice	Reduces the gene expression of IL-17 and IFN-γ in the spleen Decreases the NO level in the serum	Reduces the symptoms of encephalomyelitis. Prevents neuroinflammation	(39)
6-gingerol enriched ethanolic extract (100 mg/kg extract for 14 days)	Mercury chloride-induced heavy metal neurotoxicity in rats	Inhibits the NO, AChE, TNF-α, NF-κB, IL-1β and IL-6 in brain tissues	Improves the behavioral function	(40)
Standard extract (by CO_2_ supercritical extraction) of ginger (200 mg/kg BW)	Spared nerve injury mice model	In spinal cord samples, ginger extract reduced phosphorylated ERK and histone deacetylase 1 (HDAC1) protein levels Reduced NF-κB, IL-1β, TNF-α and IL-6	Attenuated neuropathic pain	(41)
Gingerol-enriched ginger (GEG) extract (200, 400, and 600 mg/kg BW)	Spinal nerve ligation induced neuropathic pain model in rats	Dose responsive reduction of GFAP, CD11b, IBA1, TNF-α, NF-κB, and IL-1β levels in the amygdala, frontal cortex and hippocampus	Mitigated spontaneous pain	(42)

To summarize, treatment with different sourced ginger extracts reduces p38 MAPK, pERK, HDAC1, and GFAP activity, reduces NF-κB, TNF-α, NO, and IL-1β, IL-6, CD11b, IBA1in brain tissues under different neurological dysfunctions, demonstrating significant anti-neuroinflammatory activity.

### Punica granatum L.

*Punica granatum L*. (Pomegranate, Figure 1) belongs to the Punicaceae family and is a long-lived plant grown in tropical to warm temperate climates and even in drought conditions. It is widely cultivated in Iran, India, and the Mediterranean countries such as Turkey, Egypt, Tunisia, Spain, and Morocco. Its seed is rich in oil and contains punicic acid and some phytoestrogens; its juice is enriched with tannins and flavonoids, and its bark and roots contain alkaloids. Pomegranate has been used to treat many cancers, such as prostate, lung, breast, colon, and skin. Further, it has been used to treat many cardiovascular, reproductive, central nervous systems, inflammatory disorders, osteoarthritis, rheumatoid arthritis, and fungal and bacterial infections. Several molecular mechanisms are attributed to pomegranate’s health benefits. The pomegranate juice contains ascorbic acid, citric acid, fumaric acid, and malic acid, along with trace amounts of amino acids such as proline, methionine, and valine. Juice and peel are rich in polyphenols such as tannins and flavonoids, the main ingredients for fruit’s pharmacological potential. Barks and roots contain alkaloids [reviewed in Zarfeshany et al. (43), Khadivi et al. (44), Figure 2]. Table 3 discusses the effect of pomegranate on various neuroinflammatory conditions. Supplementation with pomegranate powder (4% in diet) for 15 months activated the phosphoinositide 3-kinase (PI3K)/Akt/mammalian target of the rapamycin signaling (mTOR) pathway. It inhibited the TNF-*α*, IL-1β, inducible nitric oxide synthase (iNOS), and IL-1 transcription in brain tissues of APPsw/Tg2576 transgenic mouse model of Alzheimer’s disease (45). This treatment inhibited the overexpression of TNF-α, IL-1β, and AChE in the hippocampal tissues of scopolamine-treated rats supplemented with 200, 400, and 800 mg/kg of pomegranate seed hydroethanolic extract orally for 3 weeks, (46). Further, this treatment inhibits the release of TNF-α, IL1*β*, and iNOS in the brain of a rotenone-induced PD model of rats with an oral dose of 150 mg/kg of pomegranate extract (standardized to 40% ellagic acid polyphenol) for 20 days (47). In the APPsw/Tg2576 transgenic mouse model of AD, mice fed with 4% pomegranate in their diet for 15 months reduced the plasma levels of IL-2 and IL-3. IL-4, IL-5, IL-9, etc., and eotaxin (48). Also, pretreatment with pomegranate juice and pomegranate seed ethanolic extract increased Tyrosine hydroxylase in substantia nigra and dopamine in the striatum while reduced striatal NF-кB, CD11b, transforming growth factor-beta (TGF-*β*) and increased IL-10 and glial cell-derived neurotrophic factor (GDNF), in the striatum of a paraquat-induced mouse model of PD (49).

**Table 3:** The anti-neuroinflammatory activity of *Punica granatum.*

Dietary source	Study model	Molecular/biochemical analysis	Functional benefits	Ref
Freez-dried Fresh pomegranate powder (4% in the diet for 15 months)	APPsw/Tg 2,576 transgenic AD model of mice	Inhibited the transcription of TNF-α, IL-1β, iNOS, CCl2, and IL-1 in brain tissues Increases the expression of synaptic structural proteins in brain homogenates. Increases the protein expression of Bcl-1 and LC-3 (LC-3 type II) in the brain. Activates the-PI3K/Akt/mTOR signaling pathway	Inhibits neuroinflammation Improves the synaptic structure and functions Promotes autophagy in neurons	(45)
Pomegranate seed hydroethanolic extract (200, 400, and 800 mg/kg extract for 3 weeks)	Scopolamine-induced AD rat model	Inhibits the overexpression of TNF-α, IL-1β, and AChE in the hippocampal tissues	Ameliorates cognitive and behavioral decline	(46)
Pomegranate powder extract (standardized to 40% ellagic acid polyphenol) (150 mg/kg extract for 20 days)	Rotenone-induced Rat model of PD	Pomegranate powder extract increases the levels of dopamine, norepinephrine, 5-HT, and glutamate Inhibits the release of TNF-α, IL-1β, and iNOS Reduce the caspase-3 Reduces the AchE activity in striatal tissue homogenate	Improves locomotor activities and cognition Inhibits oxidative stress and neuroinflammation Prevents striatal neuronal apoptosis Rescues the standard histological architecture in the cerebral cortex, hippocampus, and striatum	(47)
Powdered pomegranate fruits (4% in diet for 15 months)	APPsw/Tg2576 transgenic mouse models of AD	IL-2, IL-3. IL-4, IL-5, IL-9, IL-10, and Eotaxin levels were significantly reduced in the plasma	Brain cortex and hippocampus Aβ1–40 and Aβ1–42 contents were significantly reduced	(48)
Pomegranate juice and pomegranate seed ethanolic extract	Paraquat-induced Parkinsonian-like mouse model	Both increase the level of tyrosine hydroxylase in the substantia nigra and dopamine in the striatum Reduction in the oxidative stress in the striatum. Inhibited the expression of striatal NF-кB gene, CD11b, and TGF-β Increased IL-10, GDNF and ATP in the striatum	Provided neuroprotection and maintained neuronal function	(49)

Overall, pomegranate juice/seed extract/powder activated PI3K/Akt/mTOR signaling pathway inhibits transcription of TNF-*α*, IL-1β, iNOS, CCl2, IL-1, NF-кB, CD11b, TGF-β in brain tissues and reduces the plasma levels of IL-2 and IL-3. IL-4, IL-5, etc. and eotaxin while enhancing brain IL-10 and GDNF levels.

### Emblica officinalis

*Emblica officinalis* (syn *Phyllanthus emblica Linn,* Figure 1) is commonly known as amla and is mainly grown in tropical and subtropical countries such as China, India, Indonesia and southeast Asia. It contains polyphenols like gallic acid, ellagic acid, various tannins, minerals, vitamins, amino acids, fixed oils, and flavonoids like rutin and quercetin. It has been widely used to treat several ailments, including cancer, osteoporosis, neurological disorders, hypertension, other lifestyle-related diseases and infectious disorders (reviewed in Variya et al. (50), Prananda et al. (51), Figure 2).

Administration of 40 and 80 mg/kg extract (tannins-enriched fraction) of *Phyllanthus emblica* Linn. fruit polyphenols (PEFPs) to acute paradoxical sleep deprived (SD) mice attenuate SD-induced cognitive impairment and anxiety-like behavior dose-dependently. PEFP-administered sleep-deprived animals were also shown to reduce neuronal cell injury in the cornu ammonis-3 (CA3) region and increase the dendritic spine density in the conu ammonis-1 (CA1) region of the hippocampus. PFEP also decreased the protein expression of IL-6, TNF-*α*, and IL-1*β* in hippocampal cells, indicating reduced neuroinflammation caused by the SD (52). Another study used a high-salt and cholesterol diet (HSCD) to induce neuroinflammation and cognitive impairment in rats. A tannins-enriched fraction of EO (100 and 200 mg/kg b.w) is shown to inhibit the TNF-α and IL-10, reduce the amyloid β, decrease neuronal death, and inhibit the overexpression of NF-kB in the brain (53).

Thus, administering PEFPs reduced IL-6, TNF-α, and IL-1β in hippocampal cells, inhibited the TNF-α and reduced NF-kB in the brain in different neurological disease models.

### Piper nigrum

*Piper nigrum* (Figure 1) produces black pepper, which has been extensively used in diet preparations. Geologically, it is seen in India, Malaysia, Indonesia, China, Thailand, Sri Lanka, Vietnam, Brazil and Madagascar. It is widely used in traditional medicine, perfumes, preservatives and insecticides. In conventional medicinal systems, it has been used to increase appetite to treat cough, cold, dyspnea, throat diseases, fever, dysentery, stomachache, worms, piles, inflammation, epilepsy, snake bite, rheumatism, blood circulation-related ailments etc. It contains essential oils such as oleoresin, piperine, and other compounds [reviewed in Butt et al. (54), Takooree et al. (55), Figure 2].

Piperine is the major alkaloid constituent of black pepper. In an aluminum chloride (AlCl3)-induced AD model in Sprague–Dawley rats, *Piper nigrum* methanolic extracts were supplemented daily for 3 months at a daily dose of 187.5 and 93.75 mg/kg b.w. Evaluation of the brain and serum identified that C-reactive protein (CRP), total NF-κB, and monocyte chemoattractant protein-1 (MCP-1) were significantly reduced, suggestive of amelioration of neuroinflammation with this treatment (56). In scopolamine-treated male Swiss albino mice, treatment with black pepper containing viphyllin at 50 and 100 mg/kg/b.w. for 14 days demonstrated anti-inflammatory effects by reducing the levels of COX-2, TNF-*α*, and nitric oxide synthase 2 (NOS-2) in the brain. Further, this treatment reduced the pro-brain-derived neurotrophic factor/mature brain-derived neurotrophic factor (proBDNF/mBDNF) ratio and increased the expression of tropomyosin receptor kinase B (TrkB). This treatment reduced the phosphor jun N-terminal kinase (p-JNK) and p-p38 MAPK proteins, Bcl-2-associated protein x/B-cell leukemia/lymphoma 2 protein (Bax/Bcl-2) ratio, and caspase activation in the brain at higher doses. This treatment dose-dependently improved rats’ recognition, spatial memory, and cholinergic functions (57).

In summary, different extracts of *Piper nigrum* reduced brain and serum levels of CRP, total NF-κB, and MCP-1, reduced Cox-2, TNF-α, and NOS-2 in the brain and reduced the proBDNF/mBDNF ratio and (p-JNK) and p-p38 MAPK proteins, (Bax/Bcl-2) ratio, and caspase activation in the brain at higher doses.

### Mucuna pruriens

*Mucuna pruriens* (MP, Figure 1) is commonly known as *Kapikacchu*. It is widespread in tropical and sub-tropical regions of the world. Its main constituent molecules are L-dopa, hallucinogenic tryptamines, phenols and tannins. In traditional medicines, it has been used to treat Parkinson’s disease, other neuronal disorders, arthritis, and scorpion stings and has been shown to have anti-diabetic, aphrodisiac, anti-neoplastic, anti-epileptic, and anti-microbial activities [reviewed in Lampariello et al. (58), Figure 2].

Supplementation of 750 mg/kg b.w MP seed hydroalcoholic extract for 8 weeks to rats fed with a cafeteria diet (a calorie-rich diet) reduces the food intake and body weight. MP extract also improved the obese-induced anxiety and depression-like behavior. Further, MP also rescued the obesity-induced hippocampal cellular damage and inhibited the overexpression of IL-6 in hippocampal tissues, indicating reduced neuroinflammation induced by the obesity (59). Significant behavioral abnormalities, decreased antioxidant defense, and an increase in inflammatory markers such as GFAP, iNOS, intercellular adhesion molecule (ICAM), and tumor necrosis factor-gamma (TNF-*γ*) were observed in pars compacta of substantia nigra (SNpc) of the 1-methyl-4-phenyl-1,2,3,6-tetrahydropyridine (MPTP) -induced PD mouse model. Oral administration of aqueous extract of seeds of MP (100 mg/kg b.w for 21 days) in these animals inhibited NF-κB. It increased pAkt1 activity, further preventing the apoptosis of dopaminergic neurons. Supplementation of MP also had significant antioxidant activity in the nigrostriatal region of the mouse brain. An increase in tyrosine hydroxylase and dopamine transporter immunoreactivity in SNpc of PD mice was also observed after MP treatment. The findings indicate that MP seed aqueous extract is vital in anti-neuroinflammation and restoring the biochemical and behavioral abnormalities in the MPTP-induced PD mouse model (60).

In a comparative study, a more potent neuroprotective effect of 100 mg/kg b.w MP seed ethanolic extract for 14 days than estrogen was found if administered pre and post-induction of neurotoxicity via MPTP in a mouse model of PD. MP-administered animals showed improved motor function in rotarod, footprinting, and hanging tests compared to MPTP-induced mice. Administration of MP ameliorated the MPTP-induced loss of dopaminergic neurons, increased iNOS and GFAP-positive cells, and decreased levels of dopamine, 3,4-Dihydroxyphenylacetic acid (DOPAC), and homovanillic acid in the substantia nigra region of the brain. Further, tyrosine hydroxylase positive (TH+) cells were recovered in the substantia nigra and striatal region of MP-administered MPTP-induced mice (61). In the paraquat-induced mouse model of PD, oral administration of 100 mg/kg b.w MP seeds ethanolic extract for 10 weeks is shown to reduce the NO levels and mRNA and protein levels of iNOS level in the substantia nigra region of the brain tissues indicating the reduction in the neuroinflammation. PD animals treated with MP seed extract also showed improved motor activity indicated by the rotarod test and increased TH+ cells in the striatum compared to PD-induced mice (62). In another study, MP hydroalcoholic extract with 40% levodopa, along with sesame oil once a week for 4 weeks, is shown to have anti-inflammatory activity in the rotenone-induced zebrafish model of PD. This mixture decreased the TNF-*α* and IL-1β levels in the brain tissue of the PD model, along with an increase in the dopamine levels. MP with sesame oil also improved PD-induced behavioral deficits and reduced oxidative stress in the brain (63).

It has been shown that the daily administration of 200 mg/kg b.w ethanolic extract of MP for 10 weeks attenuates the inflammation in the spinal cord injury model of rats by reducing protein expression of ectodysplasin-1 (ED1), NOS1, NF-kB, GFAP and increases the protein expression of vascular endothelial growth factor (VEGF) and neuronal nuclear antigen (NeuN) in the spinal cord. MP-treated animals also showed reduced infarct area in the spinal cord, decreased apoptosis, and improved locomotor activity compared to untreated animals (64).

Thus, supplementation of different extracts of MP reduced IL-6 in hippocampal tissues inhibited TNF-α, IL-1β, NF-κB, iNOS and GFAP-positive cells, increased pAkt1, tyrosine hydroxylase and dopamine transporter, VEGF, and NeuN in different neurological conditions.

### Clitoria ternatea L.

*Clitoria ternatea L*. (CT, Figure 1) has been shown to improve memory. It is commonly called by the names Aparajita, Girikarnika, and Vishnukrantha. Different subspecies are grown in different regions of the world. It is used in food coloring, cosmetics and insecticides. Traditional medicine uses it to treat cognitive deficits, fever, inflammation, pain, and diabetes. Several studies show that CT extract has diuretic, nootropic, antiasthmatic, anti-inflammatory, analgesic, antipyretic, antidiabetic, antilipidemic, anti-arthritic, antioxidant, and wound healing properties. It contains flavonol glycosides, phenolic aglycones, cinnamic acid, and other compounds. [reviewed in Oguis et al. (65), Escher et al. (66), Figure 2].

In a rat model of autism induced by intra-cerebro-ventricular (ICV) infusion of propionic acid, elevated levels of TNF-*α* and IL-6 were normalized by oral administration of ethanolic extract of roots of CT in rat brain tissues. Propionic acid injection also increases the activation of microglia and reactive astrogliosis by elevated expression of GFAP and CD 68 in the hippocampal region of the rat brain. Oral administration of 250 and 500 mg/kg b.w of CT ethanolic root extract for 28 days to these animals reduced the expression of these markers. It attenuated the neuroinflammation-induced apoptosis by decreasing the expression of caspase 3 in the brain tissues dose-dependently (67).

Intraperitoneal administration of 2-4 mg/kg flower aqueous extract of CT conjugated with cobalt nanoparticle (CT-Co3O4Nps) to streptozotocin (STZ)-induced diabetic rats improves memory and learning patterns. CT-Co3O4Nps administered to diabetic rats also showed reduced oxidative stress, AChE activity, and NO, IL-6, IL-1β, TNF-*α* and amyloid-β in their brain homogenate. CT-Co3O4Nps also restored the histological architecture and neuronal density in CA1, CA2, CA3, and CA4 regions and the dentate gyrus of the hippocampus in diabetic rats (68).

To summarize, oral administration of root extracts of CT reduced TNF-α, IL-1β, IL-6, CRP, and NO, inhibits activation of microglia and reactive astrogliosis, and reduces AChE activity in the hippocampus and other brain regions.

### Moringa oleifera

*Moringa oleifera* (MO) is called Shigru/ Shobhanjana / Sahijna /Sainjna/Munaga. It is common in Asia, Africa, Latin America, the Pacific Islands, the Caribbean, Florida, Madagascar, the Philippines, Central America, Cuba, Ethiopia, and Nigeria. Its leaves, pods, roots, flowers and bark have been used to treat various ailments such as glandular inflammation, headache, bronchitis, hepatitis, joint pain, kidney stones, ulcers, ear and tooth pain, skin infections, fever, to induce abortions, as laxative, tumors, arthritis, oxidative stress and insomnia along with antimicrobial and antifungal activities. *Moringa oleifera* contains several flavonoids, phenolic acids, polyphenols, phenols, glucosinolates, phytosterols, and fatty acids [reviewed in Satpathy et al. (69), Figure 2].

In a rat model of lead acetate (PbAc) induced neurotoxicity, treatment with 250 mg/kg b.w/day MO methanolic extract orally for 2 weeks showed a marked decrease in IL-1β and TNF-*α* levels. In addition, MO methanolic extract attenuated the PbAc-induced increased expression of iNOS mRNA and NF-κB p65 protein levels. The methanolic extract of MO prevented the infiltration and activation of immune cells. It inhibited the production of pro-inflammatory cytokines in cortical tissues by inhibiting the NF-κB signaling pathway (70). An effective antineoplastic drug, methotrexate, induces neurotoxicity by increasing oxidative stress and inflammation (via elevation of TNF-*α*, IL-6, and NO) in brain tissues. Additionally, co-administration of methotrexate and MO seed oil (5 mL/kg b.w) for 17 days in rats favorably alters the cerebral activities of acetylcholinesterase, antioxidant enzymes, lipid peroxidation, reduced glutathione, nitric oxide, and cytokine levels (71). The hippocampus isolated from the neurotoxin scopolamine-exposed rat showed a high NF-κB protein expression level. Twenty-eight days of pretreatment with MO seed oil (2 mL/kg b.w/day) and MO leaf aqueous extract orally (500 mg/kg b.w/day) attenuated the expression of this protein, indicating the anti-neuroinflammatory action of these products of MO (72).

Supplementation of 1, 5, and 10% of air-dried, powdered leaves of MO in the diet for 7 and 14 days to a mouse model of spatial memory deficit with scopolamine shows significant improvement in spatial memory, reduces the AChE activity along with a reduction in the TNF-*α* levels in the brain tissue homogenate. The MO-administered diseased mice also showed reduced oxidative stress and degeneration of hippocampal neurons (73). Using the APP/PS1 AD mouse model, it has been shown that oral administration of methanol extract from leaves of MO (400 mg/kg/day for 4 months) attenuates the AD-related anxiety-like behavior and hyperactivity and cognitive, learning, and memory impairments. MO administration also improved synaptic plasticity and inhibited neurodegeneration in the hippocampus. MO inhibits the neuroinflammation in APP/PS1 mouse’s hippocampus by inhibiting the protein expression of GFAP, IBA1, TNF-*α*, and IL-1β (74).

In the cobalt chloride (CoCl2) induced hypoxia model of rats, treatment with 400 mg/kg/day ethanolic extract of MO orally before or along with CoCl2 is shown to inhibit oxidative stress and CoCl2-induced depletion of 5-hydroxytryptamine (5HT), norepinephrine (NE), dopamine (DA), and gamma-aminobutyric acid (GABA) in the brain tissue. Similarly, MO-administered CoCl2 animals showed reduced expression of mRNA of hypoxia-inducible factor 1-alpha (HIF1-α), and NF-kB in hippocampal tissues. The histological alteration reduced apoptotic markers, and GFAP was also observed in the cerebral tissue of the MO-treated hypoxia model of animals (75).

In vanadium-intoxicated mice, intraperitoneal administration of 2, 5, 10 mg/kg b.w with phenolic derivative of a methanolic extract of MO leaves once in 2 days for 2 weeks showed improvement in locomotor activity. Treated animals also showed a significant reduction in the degeneration of Purkinje cells and CA1 hippocampal neurons and a reduction in the GFAP-positive cells in the corpus callosum. Further, reduced expression of Iba1 in the corpus callosum, somatosensory and retrosplenial cortex of the cerebrum were also found in MO-treated vanadium-intoxicated mice (76). In the cadmium chloride (CdCl2) induced neurotoxicity model of rats, administration of a fraction of ethanol extract of MO leaves (15 mg/kg b.w for 17 days) decreases the levels of IL-1β, IL-6, IL-8, NF-kB, and increased levels of IL-4 and IL-10 indicating the inhibition of CdCl2 induced neuroinflammation in the cerebral cortex. Further MO also inhibited apoptosis, hyperplasia, and angiogenesis in cerebral tissue by inhibiting the caspase-3, antigen Kiel 67 (Ki67), tumor protein p53 (p53), and sVEGF receptor expression (77). In the AlCl3-induced neurotoxicity model of rats, oral administration of MO leaf ethanolic extract for 28 days is shown to attenuate the AlCl3-induced impaired spatial learning and memory, suppression of superoxide dismutase (SOD), and increased NO in the brain tissues. MO also inhibited the IL-6, TNF-*α*, and attenuated the apoptosis by reducing the caspase-3 and increasing the Bcl-2 in the brain tissue and restoring normal histology of the cerebral cortex and hippocampus (78).

In the vincristine-induced peripheral neuropathy model of rats, oral administration of 250 and 500 mg/kg b.w of ethanolic extract of MO leaves for 21 days is shown to reduce the neuropathic symptoms as evidenced by hot plate response, tail flick latency, acetone spray tests, and improve the nerve conduction velocity. MO-administered neuropathy models of rats also showed reduced levels of serum IL-6, IL-1β, and TNF-*α* and reduced oxidative stress (79).

Ethanolic extract of MO leaves, when administered orally at the dose of 400 mg/kg/day 14 days before carbon tetrachloride (CCl4) induction in CCl4 induced hepatic encephalopathy mouse model, reduced the expression of toll-like receptor 4 and 2 (TLR4, TLR2), myeloid differentiation primary response 88 (MyD88), NF-κB genes and proteins in brain tissues. Thus, MO exerts its neuroprotective effect via TLR4/2-MyD88/NF-κB signaling. Additionally, MO administered to diseased animals showed suppressed oxidative stress and reduced levels of TNF-α and IL-6 in the brain tissue. Further, MO also improved memory and learning patterns by restoring the normal histological architecture of the hippocampus and cerebral cortex (80).

To summarize, different MO extracts have beneficial effects in decreasing IL-1β, I-6, TNF-*α*, iNOS, NF-κB p65, NO, GFAP, IBA1, IL-8, TLR4, TLR2, MyD88 and increasing levels of IL-4 and IL-10 suggesting that MO exerts its neuroprotective effect via TLR4/2-MyD88/NF-κB signaling in various neurological disorders.

### Phoenix dactylifera

*Phoenix dactylifera*, known as the date palm, has sweet edible fruits. It is mainly grown in Arabian countries and a few Asian locations, including India. Its fruit and seeds contain several amino acids, dietary fibers, minerals, vitamins, sugars, fatty acids and phenolic compounds. Fruit pulp and seeds are shown to have antimicrobial, antioxidant, anticancer, antidiabetic and anti-inflammatory activities (reviewed in Mahmoud et al. (81), Rahmani et al. (82), Alkhoori et al. (83), Figure 2).

Treatment with 250 and 500 mg/kg b.w of hydroalcoholic extract of dates daily for 14 days against LPS-induced sickness behavior in rats demonstrated reduced oxidative stress and neuroinflammation by decreasing the levels of IL-6 and TNF-*α* levels in the brain and improved behavior (84). In another study, 30 days of oral administration of 200 and 400 mg/kg of date seed methanolic extract resulted in a significant reversal of LPS-induced memory impairment, elevated the AChE levels, lowered the COX-2, lowered the TNF-*α* and IL-6, elevated the anti-inflammatory markers such as IL-10 and TGF-β1 in brain tissue of rats. Further, a molecular docking/modeling study shows that methanolic extracts containing several phenolic and flavonoid compounds in the date seed inhibited AChE and COX-2 (85). In the AD mouse model, APPsw/Tg2576 mouse, long-term (15 months) dietary supplementation of dates (4%) reduced several cytokines including TNF-α, IL-1β, IL-2, IL-3, IL-4, IL-5, IL-6, etc. and exotoxin activity in plasma of this transgenic AD animal model in comparison to the untreated disease model. It delayed senile plaque formation (48).

In a homocysteine-induced AD model of rats, administration of 200, 400 and 800 mg/kg of date palm extract on days 21 and 28 of induction of AD showed a significant reduction in serum level of TNF-α dose-dependently (86).

In summary, treatment with date palm ethanol extract reduces IL-6 and TNF-α levels. COX-2, increased IL-10 and TGF-β1 in the brain tissue of rats and reduced plasma cytokines TNF-α, IL-1β, IL-2, IL-3, IL-4, IL-5, IL-6, etc. and exotoxin activity in plasma of different neurological disorders.

### Curcuma longa

*Curcuma longa* (CL) Linn is commonly known as turmeric and belongs to the Zingiberaceae family. Turmeric plants are distributed throughout the tropic and subtropical regions of the world and have been used in India over 2000 years (87). It is India’s most widely used herbal product in daily food preparations. Turmeric is a sterile plant and does not produce any seeds. Its rhizome is an underground stem that possesses a potential medicinal property. The rhizomes are spices in many cuisines (88). In addition to traditional knowledge, several research works show that turmeric has antioxidant, anti-inflammatory, neuroprotective, anticancer, hepatoprotective, cardioprotective, immunomodulatory, antifertility, antimicrobial, antiallergic, anti-dermatophytic, and antidepressant properties [reviewed in Chattopadhyay et al. (89)]. In traditional medicinal systems, it has been used to treat a wide variety of disorders such as liver obstruction, jaundice, ulcers and inflammation, cough, cold, dental issues, indigestion, skin infections, blood purification, asthma, piles, bronchitis, tumor, wounds as an antiseptic [reviewed in Chattopadhyay et al. (89)]. Curcumin is its major constituent, and it has been widely researched, with>25,000 PubMed articles, and has many pharmacological uses. Even though curcumin is poorly absorbed in the intestine and with limited bioavailability, its availability, and ability to cross the blood–brain barrier have attracted many researchers to explore its possible role in neuronal disorders. It has been shown that curcumin can protect many cells in the CNS, such as astrocytes, microglia, and neurons. Several studies have shown curcumin’s beneficial effects on various parts of the CNS, such as the hippocampus, midbrain, cerebral cortex, and spinal cord. [reviewed in Fuloria et al. (90)]. The neuroprotective and anti-neuroinflammatory activity of *Curcuma longa* are provided below.

In a randomized, double-blind, placebo-controlled interventional study (*n* = 45/group) with overweight or prehypertension/mild hypertension men and women aged 50–69, the impact of supplementation of hot water extract of *C. longa* (WEC) was evaluated. Subjects consumed 900 mg WEC tablets, containing 400 μg bisacurone, 80 μg turmeronol A and 20 μg turmeronol B. Individuals were analyzed following 12 weeks of treatment for a medical outcome study (MOS) 36-item short-form health survey (SF-36) and the profile of mood states scale (POMS) along with serum inflammatory and metabolic markers. This treatment significantly improved SF-36 scores (for general health, vitality, mental health, and mental summary component) and POMS scores for positive mood states compared to placebo controls. Improvement in mental health is associated with reduced levels of systemic inflammatory markers such as C-reactive protein, TNF-*α*, IL-6, and soluble vascular cell adhesion molecule-1 and improved levels of metabolic markers. This study highlights the antiinflammatory efficacy of CL for treating overall mental health and well-being in overweight or prehypertension/mild hypertension individuals (91).

The same group evaluated the efficacy of a mixture of a hot water extract and a supercritical carbon dioxide extract of CL (CLE) in a randomized, double-blind, placebo-controlled study (*n* = 45/group) in overweight but healthy individuals aged 50 to 69 years. Subjects consumed 970 mg CLE capsules, containing 400 μg bisacurone, 100 μg turmeronol A and 100 μg turmeronol B. Individuals were analyzed following 12 weeks of treatment for a medical outcome study (MOS) 36-item short-form health survey (SF-36) and the Profile of Mood States scale (POMS) along with serum inflammatory and metabolic markers. CLE treatment significantly improved mental health scores and reduced systemic C-reactive protein and complement component 3 compared to the placebo group, highlighting the anti-inflammatory potential of CLE in controlling mental health in overweight individuals (92).

Transgenic mice Tg2576 over-expressing Aβ protein, a well-established AD model, were treated with a standardized turmeric extract (of supercritical CO_2_ extracted), HSS-888 (5 mg/mouse/day), for 6 months. This treatment significantly reduced brain levels of soluble, insoluble amyloid beta (Aβ) and phosphorylated Tau protein by ~40%, ~20% and ~ 80%, respectively, compared to untreated mice with AD phenotype. Further, microglia cultured from these treated mice demonstrated significantly reduced expression of cytokines IL-4 and IL-2 compared to untreated mice with AD phenotype. This indicates the potential importance of turmeric extract in reducing plaque burden, Tau phosphorylation, and microglia-mediated inflammation in AD (93).

In a modified Marmarou weight drop model of repetitive traumatic brain injury (rTBI), 66 male rats were administered daily with turmeric extract (with 18% curcumin) (500 mg/kg body weight) in the morning. This treatment significantly reduced the expression of TNF-*α*, GFAP, p-tau, and TAR DNA-binding protein 43 (TDP-43) in the rats exposed to rTBI compared to untreated rats. This indicates turmeric provides neuroprotection through its anti-inflammatory action (94).

To summarize, CL demonstrated beneficial effects in clinical trials for various neurological disorders. It reduced serum levels CRP l, TNF-α, IL-6, and soluble vascular cell adhesion molecule-1, complement component 3, IL-4, IL-2 GFAP, etc.

## Conclusion and future perspectives

Prevention of prolonged chronic neuroinflammation is important to avoid further complications such as systemic inflammation and progressive loss of structural and functional integrity of neurons. Established anti-inflammatory drugs cannot be used for chronic disorders due to their multiple side effects on long-term usage. At the same time, advanced biological agents are unaffordable for their continuous use. It is evident from preclinical studies that, regular use of dietary products with proven efficacy could help control chronic neuroinflammation in patients.

In this review, we have reviewed *Bacopa monnieri, Centella asiatica, Emblica officinalis, Piper nigrum, Zingiber officinale, Punica granatum, Mucuna pruriens, Clitoria ternatea, Moringa oleifera, Phoenix dactylifera* and *Curcuma longa* based on the abundance of data that demonstrated significant anti-neuro-inflammatory potentials to use for various neurological disorders regularly. As shown in Figure 2, each dietary herb reviewed in this manuscript is known to contain several bioactive molecules, and each one may possess different molecular pathways [reviewed in Siahaan et al. (95)]. Besides, these dietary herbal products possess multiple bioactive components demonstrating anti-inflammatory potential in different disease models. These data were not included in this review, as the bioactive compounds are not used individually as dietary products in regular use.

**Figure 3:**
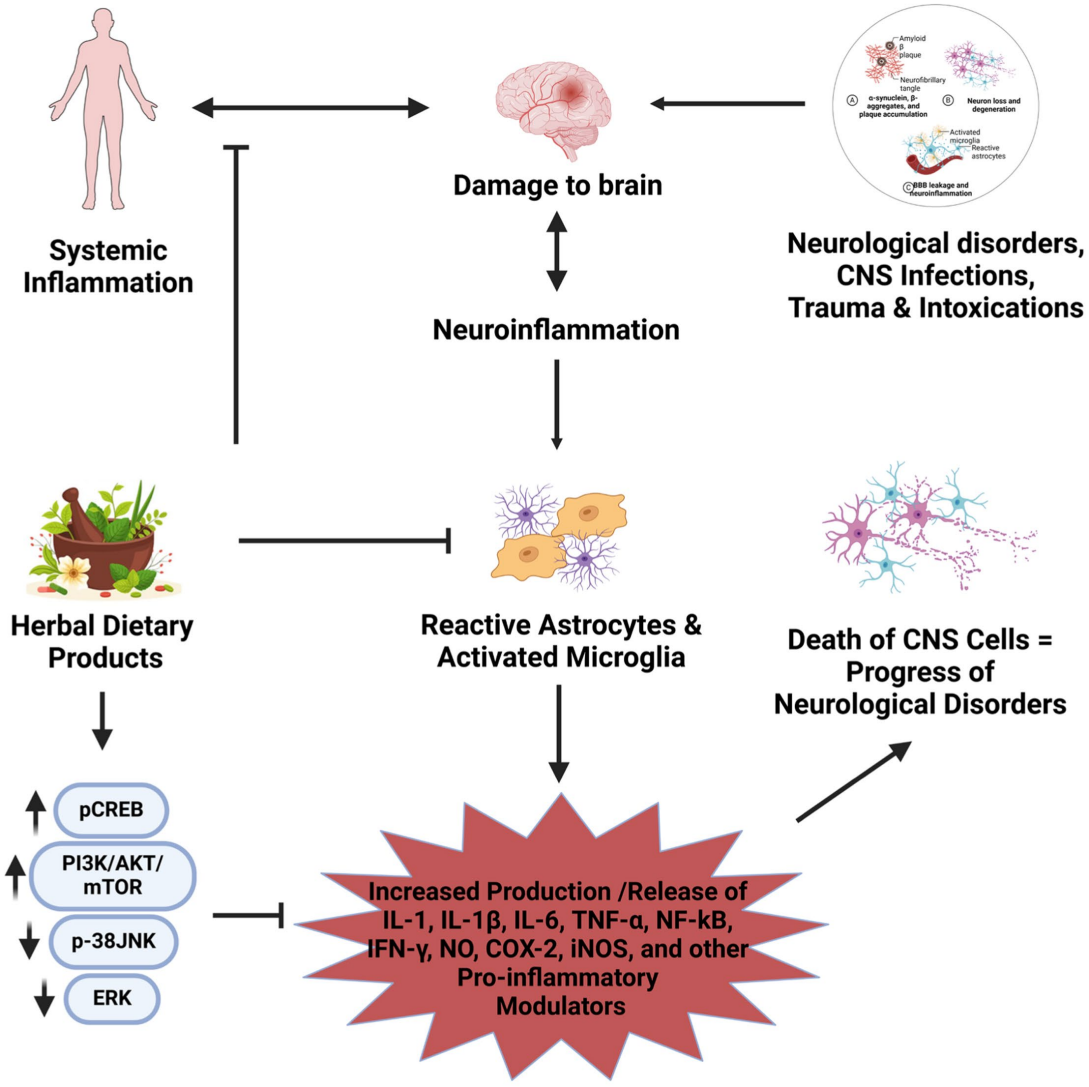
Graphical representation of the general effect of herbal dietary products on neuroinflammation and neurological disorders. In conditions of neurological disorders, accumulation of Aβ, tau, α-synuclein, neurofibrillary tangles, plaque formation, neuron degeneration, neurotrauma, or environmental toxicants, or conditions of infections could activate transmembrane pattern-recognition cell surface receptors such as TLRs, particularly in astrocytes and microglia. Activated glial cells secrete IL-1/IL-1β, IL-6, NF-κB, TNF-α, IFN-*γ*, NO, iNOS, COX-2, CCl2, etc. If unchecked, in conditions such as neurological disorders, chronic inflammation causes enormous functional damage to neurons and glial cells, including astrocytes, microglia, and oligodendrocytes. This could promote the pathogenesis of neurological disorders into an irreversible stage and also could promote systemic inflammation and damage to other organs. When administered, the constituents of the dietary herbs may interfere with these pathways and inhibit the progression of neuroinflammation, thus bringing favorable structural and functional changes in the neurons or preventing additional structural and functional damage to the CNS cells. For example, enriched bioactive components from dietary herbal products could activate pCREB or PI3K/Akt/mTOR pathways or reduce p38, JNK and ERK1/2 pathways and thereby reduce the production of NFκB, TNF-α, IL-1β, iNOS, CCl2, and IL-1, etc. Also, these dietary herbal products may act holistically with various constituents, working synergistically with the healing process using multidimensional mechanisms. This could terminate or slow down the disease propagation.

Figure 3 depicts the possible mechanisms of the actions of dietary herbal products in preventing chronic inflammation in neurological disorders. For example, in conditions of neurological disorders such as neurodegenerative diseases, accumulation of Aβ, tau, *α*-synuclein, neurofibrillary tangles, plaque formation, neuron degeneration, neurotrauma, or environmental toxicants, or conditions of infections could activate transmembrane pattern-recognition cell surface receptors such as TLRs, particularly in astrocytes and microglia. Activated glial cells secrete cytokines such as IL-1/IL-1β, IL-6, NF-κB, TNF-*α*, IFN-*γ*, NO, iNOS, COX-2, CCl2, etc. If unchecked, in conditions such as neurological disorders, chronic inflammation causes enormous functional damage to neurons and glial cells, including astrocytes, microglia, and oligodendrocytes. This could promote the pathogenesis of neurological disorders into an irreversible stage and also could promote systemic inflammation and damage to other organs. When administered, the bioactive constituents of the dietary herbs may interfere with these pathways and inhibit the progression of neuroinflammation, thus bringing favorable structural and functional changes in the neurons or preventing additional structural and functional damage to the CNS cells. For example, enriched bioactive components from dietary herbal products could activate pCREB or PI3K/Akt/mTOR or reduce p38, JNK and ERK1/2 pathways and thereby reduce the production of NF-κB, TNF-*α*, IL-1β, iNOS, and IL-1, etc. Also, these dietary herbal products may act holistically with various constituents, working synergistically with the healing process using multidimensional mechanisms. This could terminate or slow down the disease propagation.

This review identified several neuroinflammatory molecules abundantly present during different disease pathogenesis that were significantly reduced following regular supplementation of dietary herbal products. These molecules that were regulated by dietary herbal products include TNF-*α*, IL-1β, IL-4, IL-6, IL-8, IFN-*γ*, MCP-1, iNOS, COX-2, NF-kB, GFAP, IBA1, etc. in different disease models. Controlling these molecules is an essential task in minimizing chronic neuroinflammation (Table 4). In the presented studies, the regulation of these neuroinflammatory markers is highly associated with the impact of disease modification. The potential for bias in preclinical studies, including differences in study designs, dosages, experimental models and limited outcome measures may impact the generalizability of findings. However, findings from multiple studies in different neurological diseases from different groups of scientists, provide an equal possibility of neuroregulatory effects of the dietary herbal products.

The positivity and intriguing acceptance of dietary herbal products are very high globally. Multiple dietary herbal products control TNF-α, IL-1β, and IL-6, etc., in the brain, as evidenced by a plethora of preclinical data. Thus, using them for human clinical applications in their current form will be ideal. However, animal studies are performed in a controlled environment and with inbred animals with stable, uniform genetic profiles. The doses for animal experiments are usually fixed after toxicity tests and efficacy studies. However, in humans, due to several variables such as age, sex, physical and health conditions, familial history of diseases etc., uniform fixation of the dose of a herbal product is challenging. Even in traditional medicine such as Ayurveda, the dose of each medicine is fixed based on the patient’s profile.

**Table 4:** Molecular pathways regulated by different dietary herbal products.

Dietary herb	Edible parts	Molecular regulation to control neuroinflammation
*Bacopa monnieri*	Leaves	Increases CREB phosphorylation Reduces TNF-α, IL-1β, IL-6, NF-κB and MIP-1b, in different brain regions
*Centella asiatica*	Leaves and stems	Inhibits AChE activity and nitrite levels in the brain Reduces TNF-γ and IL-1β levels in the brain tissue Increase in IL-10 and MCP-1 Modulates the AMPK, MAPK, GSK-3β, Nrf2 expression Reduces the plasma levels of IL-6, IL-1β, TNF-α, IFN-γ Activates Akt and ERK1/2 pathways
*Zingiber officinale*	Rhizome	Reduces p38 MAPK, pERK, HDAC1, and GFAP activity Reduces NF-κB, TNF-α, NO, and IL-1β, IL-6, CD11b, IBA1in brain
*Punica granatum*	Fruit seeds	Activates PI3K/Akt/mTOR signaling pathway Inhibits transcription of TNF-α, IL-1β, iNOS, CCl2, IL-1, NF-кB, CD11b, TGF-β in brain tissues Reduces the plasma levels of IL-2 and IL-3. IL-4, IL-5 Enhances brain IL-10 and GDNF levels
*Emblica officinalis*	Fruits	Reduces IL-6, TNF-α, and IL-1β in hippocampus Inhibits TNF-α and reduced NF-kB in the brain
*Piper nigrum*	Dried fruits	Reduces brain and serum levels of CRP, total NF-κB, and MCP-1 Reduces Cox-2, TNF-α, and NOS-2 in the brain Reduces the proBDNF/mBDNF ratio, p-JNK and p-p38 MAPK proteins, Bax/Bcl-2 ratio, and caspase activation in the brain
*Mucuna pruriens*	Green pods and beans	Reduces IL-6 in hippocampal tissues Reduces TNF-α, IL-1β, NF-κB, iNOS and GFAP levels Increases pAkt1, tyrosine hydroxylase and dopamine transporter, VEGF, and NeuN levels
*Clitoria ternatea*	Flowers, leaves, young shoots and tender pods	Reduces TNF-α, IL-1 β, IL-6, CRP, and NO Inhibits microglia activation and reactive astrogliosis Reduces AChE activity in the hippocampus and other brain regions
*Moringa oleifera*	Pods and leaves	Decreases IL-1β, I-6, TNF-α, iNOS, NF-κB p65, NO, GFAP, IBA1, IL-8, TLR4, TLR2, MyD88 Increases IL-4 and IL-10 levels
*Phoenix dactylifera*	Fruits	Reduces IL-6 and TNF-α levels. COX-2 Increases IL-10 and TGF-β1 in the brain tissue of rats Reduces plasma TNF-α, IL-1β, IL-2, IL-3, IL-4, IL-5, IL-6
*Curcuma longa*	Rhizome	Reduces serum levels CRP l, TNF-α, IL-6, and soluble vascular cell adhesion molecule-1, complement component 3, IL-4, IL-2, GFAP, etc.

A comparative analysis is required to identify the best dietary herbal product among the bigger pool of products. This analysis should be performed in an established preclinical neuroinflammatory disease model to avoid disease-based differences in efficacy. Also, studies to identify the synergistic effects of several herbal dietary products in regulating neuroinflammation could be helpful for better controlling the progression of disease. While such synergistic effects benefit disease control, molecularly understanding their mechanisms is more complicated due to several bioactive compounds from different products. Future studies could identify the most effective herbal product and synergistic action when combined for controlling chronic neuroinflammation.

It is also essential to explore the possible adverse herb-drug interaction when dietary or non-dietary herbs are used along with other drugs to treat neurological disorders in patients (96). This is further relevant if the herb enhances/reduces the efficacy or causes adverse reactions with the main treatment used for a specific neurological disorder. For example, piperine from *Piper nigrum* could enhance the bioavailability of rosuvastatin, peurarin and docetaxel by inhibiting cytochrome P450 3A (CYP3A) and P-glycoprotein activity. This could enhance the pharmacological effects of these drugs (97, 98). Thus, interactions between drugs and the major bioactive components of herbs need to be explored using *in silico* models. Further, evaluation of single-dose/single dose, single dose/multiple doses, multiple doses/single dose and multiple doses/multiple dose combinations of herbs and drugs under *in vivo* conditions should provide a detailed impact of herb/drug interactions and the impact of these interactions on bioavailability, efficacy and adverse effects of the combinations (99, 100).

Clinical studies are warranted to identify and validate the clinical efficacy of these herbal dietary products against controlling chronic neuroinflammation in various neurological disorders. However, it is harder to demonstrate pharmacokinetics and pharmacodynamics data due to their complex composition with multiple components. Further, the dosage limit should be standardized to ensure biochemical consistency, safety and efficacy of every herbal dietary product (101). Differential bioavailability of the bioactive components from the dietary herbal product is common. It must be evaluated to ensure that the key components expected to drive the efficacy are bioavailable. Also, if used as a commercial product, pharmacovigilance issues such as product quality, adulteration, batch-to-batch variability in the number of active constituents, differences in the extraction process, macro and microscopic properties, presence of heavy metals, pesticide residues and microbial contaminants need to be intensely evaluated before their clinical recommendations.

To conclude, as commonly used in the diet, dietary herbal products are proven nondetrimental to health while providing exceptional benefits for controlling neuroinflammation. A way forward is undertaking well-planned molecular studies to identify their direct and off-targets. Future research that verifies and promotes herbal dietary products for controlling chronic neuroinflammation will be welcomed. This could reduce the long-term dependency on currently used anti-inflammatory drugs that have serious side effects and economic burdens.
